# Fully Automatic System for Accurate Localisation and Analysis of Cephalometric Landmarks in Lateral Cephalograms

**DOI:** 10.1038/srep33581

**Published:** 2016-09-20

**Authors:** Claudia Lindner, Ching-Wei Wang, Cheng-Ta Huang, Chung-Hsing Li, Sheng-Wei Chang, Tim F. Cootes

**Affiliations:** 1 Centre for Imaging Sciences, The University of Manchester, Oxford Road, M13 9PT Manchester, United Kingdom; 2 Graduate Institute of Biomedical Engineering, National Taiwan University of Science and Technology, Taiwan; 3 NTUST Centre of Computer Vision and Medical Imaging, National Taiwan University of Science and Technology, Taiwan; 4 Department of Information Management, Oriental Institute of Technology Taiwan; 5 Orthodontics and Dentofacial Orthopedics Division, Dental Department, Tri-Service General Hospital, Taiwan; 6 School of Dentistry & Graduated Institute of Dental Science, National Defense Medical Center, Taiwan

## Abstract

Cephalometric tracing is a standard analysis tool for orthodontic diagnosis and treatment planning. The aim of this study was to develop and validate a fully automatic landmark annotation (FALA) system for finding cephalometric landmarks in lateral cephalograms and its application to the classification of skeletal malformations. Digital cephalograms of 400 subjects (age range: 7–76 years) were available. All cephalograms had been manually traced by two experienced orthodontists with 19 cephalometric landmarks, and eight clinical parameters had been calculated for each subject. A FALA system to locate the 19 landmarks in lateral cephalograms was developed. The system was evaluated via comparison to the manual tracings, and the automatically located landmarks were used for classification of the clinical parameters. The system achieved an average point-to-point error of 1.2 mm, and 84.7% of landmarks were located within the clinically accepted precision range of 2.0 mm. The automatic landmark localisation performance was within the inter-observer variability between two clinical experts. The automatic classification achieved an average classification accuracy of 83.4% which was comparable to an experienced orthodontist. The FALA system rapidly and accurately locates and analyses cephalometric landmarks in lateral cephalograms, and has the potential to significantly improve the clinical work flow in orthodontic treatment.

Cephalometric radiography is commonly used as a standard tool in orthodontic diagnosis and treatment planning as well as in corrective and plastic surgery planning. Marking of anatomical landmarks of the skull and surrounding soft tissue in lateral cephalograms is an essential part of the diagnosis and planning process. Cephalometric landmarks are used for a number of orthodontic analyses (e.g. Schwarz, Steiner, Ricketts) where several linear and angular measurements are calculated from their positions^1^. The accuracy with which the landmarks are located has a direct impact on the results of the performed analyses and resulting treatment decisions.

Identifying cephalometric landmarks in lateral cephalograms is a challenging problem. The skull is a highly complex 3D object which in a cephalogram is projected onto a single 2D plane, leading to overlapping structures. Furthermore, facial asymmetry, head positioning variations during image acquisition, and radiographic distortion cause the left and right outlines to not be perfectly superposed, leading to duplicated structures. This, combined with individual anatomical variation, in particular in pathological cases, makes it very difficult to reliably position cephalometric landmarks^2^.

Currently in clinical practice, cephalometric landmark positions are identified manually or semi-automatically which is very tedious, time-consuming and prone to inconsistencies within and across orthodontists. Varying levels of orthodontic training and experience may have an impact on *inter*-observer variations, and time-constraints and other commitments may have an impact on *intra*-observer consistency^3^.

A computerised system for automatic cephalometric landmark identification would help to overcome time-constraints and inconsistencies within and across observers. Furthermore, given the landmark positions, in today’s orthodontic software packages the landmark-based linear and angular measurements are automatically calculated. Therefore, if a computerised system was able to locate cephalometric landmarks with sufficient accuracy then this would have the potential to significantly improve the clinical work flow in orthodontic treatment.

Over the last three decades, several attempts had been made to create such a computerised system^4^,^5^,^6^,^7^,^8^,^9^,^10^,^11^. However, due to the complexity of this problem, the developed methods were unable to compete with manual landmark identification. In recent years, a number of IEEE International Symposium on Biomedical Imaging (ISBI) Grand Challenges were organised on this topic to encourage the development of better algorithms. The results are summarised in refs 12 and 13 and provide a benchmark for any future development.

Preliminary results of our approach were presented at the 2015 ISBI Grand Challenge in Dental X-ray Image Analysis, where our system was awarded the first prize^13^,^14^. Supplementary Table S1 gives a quantitative comparison of all seven techniques that were submitted to the 2014 and 2015 ISBI Grand Challenges when tested on the same dataset of 100 images. This shows that our methodology achieved an average point-to-point error of 1.66 mm compared to errors ranging from 1.85 mm to 2.85 mm for all other techniques, demonstrating that our method performed significantly better than any of the other six techniques (*p* < 0.0001). In this paper, we expand on our previous results in various ways: (*i*) we use a large dataset of 400 images to generate and test the system; (*ii*) we show significant improvements in performance; (*iii*) we show extensive in-depth experimental results; and (*iv*) we analyse the all-important link to the performance of human observers (i.e. clinical experts).

The purpose of this study was to develop and validate a fully automatic landmark annotation system for the accurate localisation of cephalometric landmarks in lateral cephalograms and its application to the classification of skeletal malformations.

## Materials and Methods

Ethical approval (IRB Number 1-102-05-017) for this study was obtained from the research ethics committee of the Tri-Service General Hospital in Taipei, Taiwan, which waived the requirement to obtain informed consent from all subjects. All experiments were carried out in accordance with the approved guidelines.

### Dataset

Lateral cephalograms were available from 400 subjects (mean age: 27.0 years; age range: 7–76 years; 235 females, 165 males). All cephalograms were acquired in TIFF format with a Soredex CRANEXr Excel Ceph machine (Tuusula, Finland) using Soredex SorCom software (3.1.5, version 2.0). The image resolution was 1935 × 2400 pixels with a pixel spacing of 0.1 mm.

**Figure 1:**
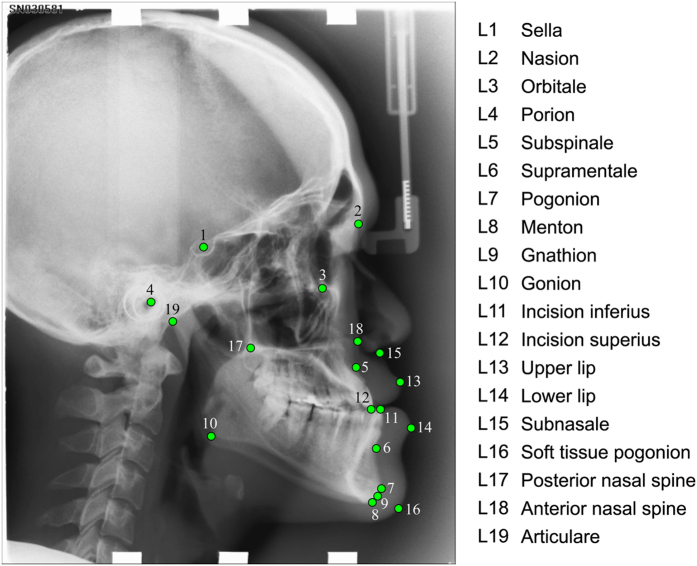
Cephalogram annotation example showing the 19 landmark positions used in this study. A description of all landmarks is given in Supplementary Table S2.

All images were annotated (“traced”) independently by two clinical orthodontists (with six years and 15 years of experience, respectively), yielding a manual annotation of 19 cephalometric landmark positions as shown in Fig. 1. All landmarks were chosen to mark common structures used in cephalometric evaluations such as Steiner Analysis or Wits Appraisal^15^. The amount of shape variation exhibited in the dataset as outlined by the 19 landmarks is shown in Supplementary Figure S3. For a random subset of 150 images, two sets of manual annotations per orthodontist were available. Based on the manually placed landmark positions, eight clinical parameters for the diagnosis of skeletal malformations were defined as listed in Table 1^16^,^17^,^18^,^19^,^20^,^21^,^22^.

### Fully Automatic Landmark Annotation System

We have recently developed a fully automatic landmark annotation (FALA) system to rapidly and accurately place a number of landmark points along the outline of the proximal femur on pelvic radiographs^23^. For this study, the FALA system was modified and improved to enable the identification of cephalometric landmarks in lateral cephalograms.

**Figure 2:**
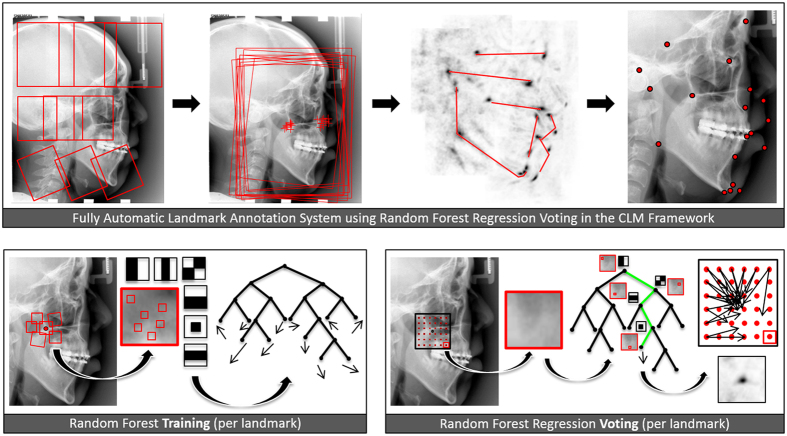
Schematic overview of the FALA system as described in refs 23 and 25, applied to lateral cephalograms.

The FALA system follows a machine learning approach where Random Forest regression-voting is used both to detect the position, scale and orientation of the skull (similar to Hough Forests^24^) and then, in the Constrained Local Model framework (RFRV-CLM), to locate the individual landmarks^25^. The first step makes the system robust to any variations in image acquisition, and the second step allows for the accurate placement of all 19 landmark points. Full details are given in refs 23 and 25, here were summarise the approach. Figure 2 gives a schematic summary.

#### Random Forest regression-voting

Random Forests (RFs)^26^ describe an ensemble of decision trees trained independently on a randomised selection of features. We train RF regressors from sets of images, each of which is annotated with landmark points ***x***. The region of interest of the image that captures all landmark points of the object is re-sampled into a standardised reference frame. For every landmark point in ***x***, a set of patches is sampled at a set of random displacements ***d***_*i*_ from the true position in the reference frame. To achieve robustness to variations in scale and orientation, patches are sampled at a range of angles and scales. Features *f*_*i*_(***x***) are extracted from each patch, and a regressor *R*(***f***(***x***)) is trained to predict the most likely position of the model point relative to ***x***. We use Haar features^28^ as they have been found to be effective for a range of applications and can be calculated efficiently from integral images. For each landmark, one RF is trained to vote for (i.e. predict) the likely position of that landmark. During search, all trees in the RF will cast independent votes to make predictions on the landmark’s position.

#### Object detection

To detect the object of interest in the image, we apply RF regression-voting as above with a single *pseudo* landmark which is defined by the centre of a patch, or reference frame, that encompasses all 19 landmark points. The RF regressor is applied across the image in a sliding window approach at a range of scales and orientations, casting votes for the centre of the reference frame into an accumulator array; using the parameters identified in ref. 23. Voting peaks will provide candidates for the likely position, orientation and scale of the object in the image. The latter is used to provide a first estimate of all landmark points which is then refined using the RFRV-CLM approach as summarised below.

#### Constrained Local Models

CLMs combine global shape constraints with local models of the pattern of intensities. Based on a number of landmark points in a set of images, a Statistical Shape Model (SSM) is trained by applying principal component analysis to the aligned shapes^27^. This yields a linear model of shape variation which represents the position of each landmark point *l* using 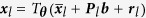 where 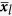 is the mean position of the point in a suitable reference frame, ***P***_*l*_ is a set of modes of variation, ***b*** are the shape model parameters, ***r***_*l*_ allows small deviations from the model, and *T*_*θ*_ applies a global transformation (e.g. similarity) with parameters *θ*.

#### RF regression-voting in the CLM framework

For a new image, given an initial estimate of the pose of the object (e.g. from an object detection step), the region of interest of the image is re-sampled into the reference frame, an area around each landmark point is searched and the relevant feature values at every position are extracted. These will be used for the trees in the RFs to vote for the best position in an accumulator array, yielding a 2D histogram of votes ***V***_*l*_ per landmark. An SSM is then used to regularise the output of the individual landmark predictors so that the resulting shape is consistent with that observed across the training data. We seek the shape model and pose parameters {***b***, ***θ***} that maximise the number of votes over all *k* landmark points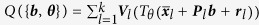, and apply the technique described in ref. 25 to solve this optimisation problem.

The RFRV-CLM search is applied in a coarse-to-fine two-stage approach that first uses a lower resolution of the reference frame to roughly estimate the position of every landmark point and then refines the results using a higher resolution reference frame. That is, based on the available manual annotations two RFRV-CLMs were trained. We apply 10 search iterations using the lower resolution RFRV-CLM and a single search iteration to refine the point positions using the higher resolution RFRV-CLM, after which the search terminates and the system outputs a single position for every landmark point. The system was developed in C++.

The accuracy and robustness of the FALA system in placing the cephalometric landmarks was analysed using four-fold cross-validation experiments. All reported results were averaged over all four runs.

### Fully Automatic Classification of Skeletal Malformations

The output of the FALA system (i.e. the fully automatically detected cephalometric landmark positions) was used to automatically determine the presence or absence of skeletal malformations as defined by the clinical parameters in Table 1. For this purpose, several measurements between landmark positions were automatically calculated, and every subject was automatically classified into one of three to five groups (C1, C2, C3, C4, and C5) for each of the eight clinical parameters. All calculations were done using custom code developed in Python.

To evaluate the performance of the fully automatic classifications, we compared them to the classifications obtained from the manual ground truth annotations.

### Statistical Analysis

The results of the performance analysis of the FALA system are reported as point-to-point errors which give the absolute distance between the manual ground truth annotations and the automatically identified landmark positions. We defined the *landmark-specific* point-to-point error for landmark *l* as 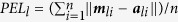 and the *image-specific* point-to-point error for image *i* as 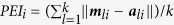 with *n* being the number of images and *k* being the number of landmarks for manual and automatic landmark positions ***m***_*l*_ and ***a***_*l*_ respectively. The overall average point-to-point error was defined as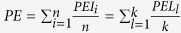. All *PEL* and *PE* are reported ± standard error (SE). All *PEI* are presented via cumulative density functions (CDFs) which give the proportion of tested images that achieved a certain average precision range. We also report the successful detection rate (*SDR*) which gives the percentage of images for which a landmark *l* was located within a precision range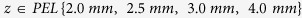: 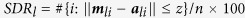. All point-to-point errors were calculated using custom code developed in Matlab R2014a. All CDF curves were calculated using custom code developed in C++ and plotted using Gnuplot.

For evaluation of the automatically obtained cephalometric classification results, we report the successful classification rate (*SCR*) which gives the percentage of accurately classified images per clinical parameter: 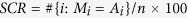 with manually and automatically classified groups *M*_*i*_ and *A*_*i*_ respectively. We present the results using confusion matrices. All classification errors were calculated using custom code developed in Matlab R2014a.

## Results

### Improvement of the Annotation System

The FALA system is based on methodology to locate points on a continuous line along a bone contour. Identifying cephalometric landmarks poses a somewhat different problem as most points occur at locally isolated positions with a lack of clearly identifiable features at the landmark’s position. Based on preliminary results in ref. 14, we investigated whether the parameter settings of the FALA system could be improved to enable the identification of isolated landmarks.

For the state-of-the-art femur annotation results in ref. 23, the parameters of the FALA system were optimised using extensive experimental evaluation. Although we have seen that the methodology is generally insensitive to most of the parameters, considering the problem of cephalometric landmark identification suggested that a re-evaluation of some of the parameters may lead to an improvement in performance. We therefore performed a series of experiments to analyse the impact on performance of varying several parameters for the high resolution refinement stage: (*i*) the size of the sampled patches (*ps*)–the larger the patch the more information about the appearance at the landmark’s position can be encoded with the features extracted from the patch; (*ii*) the range around each landmark from where patches are sampled during training (*tr*) – the larger the training sampling range the more appearance information about the environment of the landmark can be encoded; and (*iii*) the range around each landmark from where patches are sampled during searching (*sr*) – the larger the search range the more information from the area around the estimated landmark position can be included. Depending on the application area, increasing or decreasing these parameters might be beneficial or distracting (see also discussion).

**Figure 3:**
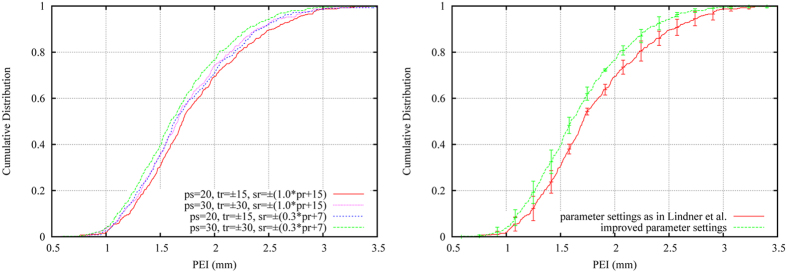
Cumulative distribution of image-specific point-to-point results for parameter optimisation of the FALA system: (left) comparing different combinations of patch size (*ps*), training range (*tr*) and search range (*sr*); and (right) showing the difference in performance between the original^23^ and the improved parameter settings. Error bars show the 95% confidence interval across the four cross-validation experiments.

We generated and evaluated various FALA systems with a range of parameter settings and compared their performance to a FALA system that used the exact same parameters as suggested in ref. 23. All systems were generated and evaluated using the annotations of the senior doctor as ground truth for both training and testing (if not stated otherwise). As in ref. 23, the reference frame width for the refinement stage was 500. The results are summarised in Fig. 3 and show that the performance of the FALA system as presented in ref. 23 can be improved for the task of cephalometric landmark identification by (*i*) increasing *ps* from 20 to 30, (*ii*) increasing *tr* from ±15 to ±30, and (*iii*) decreasing *sr* from ±(1.0*pr + 15) to ±(0.3*pr + 7) with *pr* giving the range of the landmark’s position across the aligned training data. Increasing/decreasing any of these parameters further did not lead to any improvements.

Supplementary Figure S4 visualises the voting results of the RF landmark predictors for both the original parameter settings as in ref. 23 and our improved parameter settings, demonstrating the more refined voting results for the latter.

The FALA system with the improved settings was used for all experiments described below.

### Fully Automatic Annotation Results

The FALA system achieved a PE of 1.7 ± 0.02 mm and a PEI within 2.5 mm for 95% of all 400 images. In comparison, based on all 400 annotated images, the manual *inter*-observer variability between both doctors gave a PE of 2.2 ± 0.03 mm and a PEI below 3.1 mm for 95% of all 400 images. Using the subset of 150 images, the *intra*-observer variability was 1.7 ± 0.01 mm for doctor1 (senior doctor) and 0.9 ± 0.01 mm for doctor2 (junior doctor).

**Figure 4:**
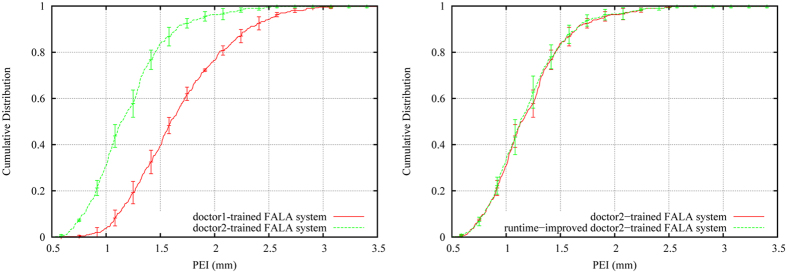
Cumulative distribution of image-specific point-to-point results of the FALA system tested on 400 images: (left) demonstrating the impact of the quality of the training data on performance; and (right) demonstrating that the runtime-improved system does not lead to any decrease in performance. Error bars show the 95% confidence interval across the four cross-validation experiments.

We repeated the experiments using the doctor2 annotations as ground truth for both training and testing. Figure 4 (left) shows the comparison of the FALA system trained on the doctor1 vs doctor2 annotations, demonstrating that the doctor2-trained system significantly outperforms the doctor1-trained system. This highlights the impact of the quality of the training data on the performance of the system.

**Figure 5:**
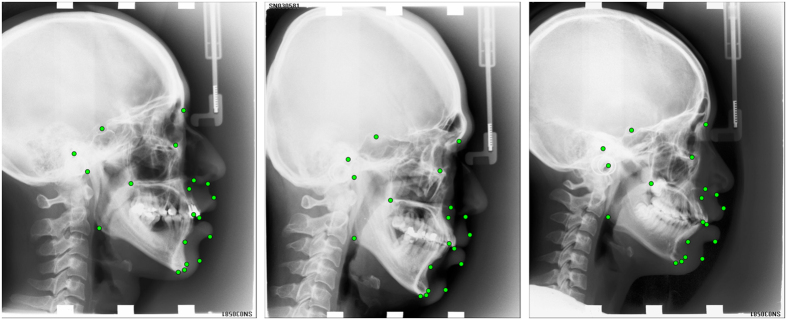
Fully automatic annotation results of the runtime-improved doctor2-trained FALA system (sorted by PEI percentiles): (left) 50%ile/median: PEI = 1.1 mm; (middle) 95%ile: PEI = 1.8 mm; and (right) 99%ile: PEI = 2.4 mm.

For the results in Fig. 4 (left), the fully automatic search of the doctor2-trained FALA system took on average 24 seconds. To improve the runtime, we optimised the search such that the system would not continue searching an image once it has found an annotation that it considers “good enough”. We describe the latter by the average distance of the RF predictions for each landmark position, and assume predictions within on average 4 pixels in the refinement reference frame to be sufficient. Figure 4 (right) compares the doctor2-trained system with the runtime-improved doctor2-trained system. Both systems achieved very similar performance, yielding a PE of 1.2 mm and a PEI within 1.8 mm for 95% of all 400 images. The runtime-improved FALA system searched an image in on average less than 3 seconds. Figure 5 visualises the median, 95%ile and 99%ile results of these experiments.

Table 2 summarises the landmark-specific annotation results for the runtime-improved doctor2-trained FALA system. On average 84.7%/96.3% of all landmarks (across all images) were located within a 2.0 mm/4.0 mm precision range. This is significantly better than the SDRs of the manual *inter*-observer analysis where only 62.1%/85.0% were within a 2.0 mm/4.0 mm precision range.

Table 2 also demonstrates for which landmarks the system performed best/worst. For L10, the FALA performance was significantly worse for all precision ranges which is in line with the manual *inter*-observer errors for L10 with a PEL of 2.69 ± 0.12 mm and SDRs of 50.25%, 57.00%, 65.25% and 79.75% for precision ranges 2.0 mm, 2.5 mm, 3.0 mm and 4.0 mm, respectively. The largest manual *inter*-observer errors were found for L16 with a PEL of 6.57 ± 0.18 mm. The FALA performance for L16 was significantly better with a PEL of 1.23 ± 0.06 mm for the doctor2-trained system and a PEL of 3.87 ± 0.20 mm for the doctor1-trained system.

### Fully Automatic Classification Results

We used the automatically identified landmark positions from the cross-validation experiments to calculate and classify the clinical parameters specified in Table 1. The results of comparing the automatic (i.e. from the doctor2-trained runtime-improved FALA system located landmarks) to the ground truth (i.e. from the doctor2 manually placed landmarks) classifications are shown in Table 3. The automatic classification achieved an average SCR of 78.4% (over all classes), and an overall average classification accuracy of 83.4% (over all subjects). The worst average SCR was achieved for FHI (63.5%), and the best for APDI (89.3%). Table 3 demonstrates that overall misclassification rates were low except for FHI (80.0%, C2 misclassified as C1) and SNA (30.7%, C3 misclassified as C1).

For comparison, Table 4 gives the results of the *inter*-observer errors between the two ground truth classifications, yielding an average SCR of 75.4% and an overall average classification accuracy of 79.7%. This shows that the automatic classification performance was within the inter-observer variability between two manual observers.

Furthermore, the misclassification patterns of the manual classifications were similar to the ones of the FALA system. This may reflect the general difficulty of reliably classifying some of these very narrow-ranged parameters (e.g. less than 3° difference between three classes), or may demonstrate that the FALA system “inherited” some of the inaccuracies from the manual training data. The latter is indicated, for example, by FHI – a parameter whose definition depends on the worst performing landmark L10 – showing the highest misclassification rates. In addition, the FALA system performed significantly better for SNA whose definition includes L5 which was identified by the FALA system with a PEL of 1.44 ± 0.05 mm but had an *inter*-observer PEL of 2.89 ± 0.15 mm. The superior classification performance of the FALA system for SNA might be due to the system obtaining more consistent results.

Supplementary Table S5 also gives the classification results for the doctor1-trained FALA system which achieved an average SCR of 75.8% and an overall average classification accuracy of 79.8%. Similar to the annotation results, this shows that the doctor2-trained system outperforms the doctor1-trained system. However, both systems are within the range of the manual inter-observer variability.

## Discussion

We have modified a system previously developed to identify landmarks along bone contours to successfully locate (isolated) cephalometric landmarks. When identifying landmarks along bone contours the difficulty lies in accurately outlining the correct contour as in 2D radiographs overlaying structures may lead to a range of bone contours in close proximity to the contour of interest. In cephalometric images, the challenge for most landmarks is slightly different as the focus is on identifying the correct landmark position independently of whether this is on a bone contour or not. Our experiments showed that increasing both the patch size and the training sampling range is beneficial for the identification of cephalometric landmarks. This is likely to be because a larger patch size allows for more meaningful patches covering more structure around the landmark of interest (beyond the bone contour at the landmark’s position), and the increased training sampling range allows the system to learn more about the environment of the landmark. In contrast, for the application to bone contours (e.g. proximal femur) a larger patch size as well as a larger training sampling range would not include much additional information as for most landmarks there is not much appearance variation either side of the bone contour. Furthermore, in the case of bone contour annotation, due to the limited information that is available locally the search range was chosen relatively large so as to include additional information from outside the object of interest (e.g. pelvis) to guide the search – which has been shown to improve performance^23^. However, cephalograms reflect the complexity of the skull and show a lot of structural information and variation. Therefore, for cephalometric landmarks, a large search range can lead to conflicting information in the voting arrays (see e.g. Supplementary Figure S4), and we found a decreased search range to be beneficial for this application. The developed FALA system for cephalometric landmark identification, hence, combines an increased patch size, an increased training sampling range, and a decreased search range to show improved performance.

The runtime-improved doctor2-trained FALA system achieved a PE of 1.2 mm and a PEI of 1.8 mm for 95% of all 400 images. Our reported SDR of 84.7% for the clinically accepted 2.0 mm precision range is an improvement of almost 10% when compared to the 74.8% reported in ref. 13. In our experiments we found the landmark localisation performance of the system to be comparable to a well-trained and experienced orthodontist. Overall, our results suggest that the accuracy of the system lies within the inter-observer variability between two clinical experts. Compared to our preliminary results in refs 13 and 14, this work shows significant improvements in landmark localisation performance (PE of 1.66 mm vs 1.20 mm) which is attributable to the efficient utilisation of a larger training dataset and more consistent manual landmark placing. This study highlights the importance of the quality of the training data and demonstrates that the performance of our methodology directly benefits from improved ground truth data. We believe this to be the most accurate and robust fully automatic cephalometric landmark detection system yet reported. Furthermore, our results show that using the FALA output we can achieve similar accuracy in automatically classifying clinical orthodontic parameters to the estimated error between two clinical experts.

In terms of landmark-specific analysis, the FALA performance was worse for L10. However, the results were within the inter-observer variability between two medical experts and were also in line with previously reported results^13^. This suggests that it is generally very difficult to place L10 accurately and consistently on lateral cephalograms. The large manual *inter*-observer variation for L16 is likely due to the less specific definition of this landmark in clinical practice, as discussed in ref. 12. The better performance of the doctor2-trained FALA system for L16 can be explained by the system replicating the quality of the training data, and hence learning the annotation approach and consistency given by the manual annotations. This is also supported by L16 having the largest error amongst all landmarks for the system trained on the less consistent doctor1 annotations.

The difference in performance between the doctor1-trained and the doctor2-trained systems does not only show that the FALA system replicates the manual annotation accuracy but also highlights that there may be large annotation variations in clinical practice^3^. The latter may be due to different levels of training or experience, and other circumstances such as time pressure and physical condition (e.g. fatigue). In contrast, the FALA system is deterministic and will *always* give the same result for the same image.

As demonstrated in ref. 13, our methodology significantly outperforms alternative cephalometric landmark detection methods. All techniques submitted to the 2014 and 2015 ISBI Grand Challenges followed a supervised learning approach with RFs being the main method of choice^13^. We believe that the superior performance of our system results from a combination of: (*i*) applying regression-voting instead of classification; (*ii*) having a separate and efficient object detection step to reduce the noise on the votes; (*iii*) integrating votes from the area around each landmark; (*iv*) optimising the voting results using an SSM; and (*v*) following a coarse-to-fine search strategy.

All experiments were performed in a VMware running Ubuntu 14.04 LTS with a quad-core 3.6 GHz CPU and 4 GB RAM. Our system can be trained and run on an average PC or laptop without the requirement for any specialist hardware. The training of a runtime-improved FALA system as used in the cross-validation experiments took about 12 hours (1 hr for the object detection, 4.5 hrs for the low resolution RFRV-CLM and 6.5 hrs for the high resolution RFRV-CLM). The runtime-improved FALA system searched a single image in on average less than 3 seconds without the need for any GPU acceleration or parallel computing, though the latter could be implemented to further improve the runtime of the system.

Our study has several limitations. The FALA system replicates the manual annotation approach, and although the system may be more accurate than the manual annotations (e.g. by learning to average out errors) the accuracy to which the system can be evaluated is limited by the manual annotation accuracy. In addition, albeit the underlying methodology is independent of the number of landmarks used, the system would need to be retrained to include additional landmarks. Furthermore, the performance of the system depends on (*i*) the quality of the training data; (*ii*) the size of the training dataset; and (*iii*) the shape and appearance variation exhibited in the training data (e.g. age, type and degree of malformations). However, if the FALA system was trained using representative and consistent training data then it has the potential to outperform clinical experts as demonstrated by the performance of the doctor1-trained vs doctor2-trained systems.

In conclusion, we have developed and validated computer software for fully automatic cephalometric evaluation that can rapidly and accurately locate cephalometric landmarks in lateral cephalograms, and have demonstrated that the latter can be used for the automatic classification of skeletal malformations. The FALA system shows great promise for application in orthodontic software solutions to fully automatically conduct cephalometric analyses. Its deterministic properties may also contribute to the standardisation of the position of landmarks with less well-defined positions. Future work will include extending the FALA system to search for additional cephalometric landmarks. Based on our findings, it would also be interesting to investigate the correlation between high quality (i.e. consistent and accurate) annotations and clinical outcome. The latter may shed some light on the clinical importance of the quality of the annotations as well as on the validity of the clinically accepted 2.0 mm precision range.

The runtime-improved FALA system trained on all 400 images and doctor2 annotations is freely available for research purposes via www.bone-finder.com. Furthermore, all images and annotations for both the junior and senior doctors as well as code to generate the classifications and evaluate performance are available from https://figshare.com/s/37ec464af8e81ae6ebbf.

## Additional Information

**How to cite this article**: Lindner, C. *et al*. Fully Automatic System for Accurate Localisation and Analysis of Cephalometric Landmarks in Lateral Cephalograms. *Sci. Rep*. **6**, 33581; doi: 10.1038/srep33581 (2016).

## Supplementary Material

Supplementary Information

## Figures and Tables

**Table 1:** Overview of eight clinical measurements and their classifications used in the automated cephalometric evaluation^16^,^17^,^18^,^19^,^20^,^21^,^22^.

	ANB^1^	SNB^2^	SNA^3^	ODI^4^	APDI^5^	FHI^6^	FMA^7^	MW^8^
C1	3.2–5.7°	74.6–78.7°	79.4–83.2°	68.4–80.5°	77.6–85.2°	0.65–0.75	26.8–31.4°	2–4.5 mm
C2	>5.7°	<74.6°	>83.2°	>80.5°	<77.6°	>0.75	>31.4°	=0 mm
C3	<3.2°	>78.7°	<79.4°	<68.4°	>85.2°	<0.65	<26.8°	<0 mm
C4	—	—	—	—	—	—	—	>4.5 mm
C5	—	—	—	—	—	—	—	0<,<2 mm

^1^ANB: angle between A-point (L5), nasion (L2) and B-point (L6).

^2^SNB: angle between sella (L1), nasion (L2) and B-point (L6).

^3^SNA: angle between sella (L1), nasion (L2) and A-point (L5).

^4^Overbite depth indicator (ODI): sum of the angle between the lines from L5 to L6 and from L8 to L10, and the angle between the lines from L3 to L4 and from L17 to L18.

^5^Anteroposterior dysplasia indicator: sum of the angle between the lines from L3 to L4 and from L2 to L7, the angle between the lines from L2 to L7 and from L5 to L6, and the angle between the lines from L3 to L4 and from L17 to L18.

^6^Facial height index: ratio of the posterior face height (distance from L1 to L10) to the anterior face height (distance from L2 to L8).

^7^Frankfurt mandibular angle: angle between the lines from sella (L1) to nasion (L2) and from gonion (L10) to gnathion (L9).

^8^Modified Wits Appraisal: ((*x*_*L*12_−*x*_*L*11_)/|*x*_*L*12_−*x*_*L*11_|)||*x*_*L*12_−*x*_*L*11_||.

**Table 2:** Landmark-specific annotation results for the runtime-improved doctor2-traianed FALA system: landmark-specific point-to-point errors (PEL) and successful detection rates (SDR) for 2.0 mm, 2.5 mm, 3.0 mm and 4.0 mm precision ranges.

Landmark	PEL ± SE (mm)	SDR (%)			
		2.0 mm	2.5 mm	3.0 mm	4.0 mm
Sella (L1)	0.80 ± 0.05	96.75	98.50	98.75	99.25
Nasion (L2)	1.06 ± 0.06	85.00	90.00	91.25	96.50
Orbitale (L3)	1.24 ± 0.06	78.75	84.75	89.50	95.50
Porion (L4)	1.64 ± 0.10	79.25	83.50	86.50	89.75
Subspinale (L5)	1.44 ± 0.05	75.50	85.25	91.75	95.75
Supramentale (L6)	1.26 ± 0.05	83.00	89.50	94.50	98.75
Pogonion (L7)	1.00 ± 0.03	91.50	95.50	98.25	100.00
Menton (L8)	0.84 ± 0.03	94.75	97.50	98.75	99.25
Gnathion (L9)	0.80 ± 0.03	97.00	99.00	99.50	99.50
Gonion (L10)	2.69 ± 0.12	50.25	57.00	65.25	79.75
Incision inferius (L11)	0.89 ± 0.06	89.25	91.00	94.25	97.50
Incision superius (L12)	0.65 ± 0.05	92.25	93.50	95.25	98.50
Upper lip (L13)	1.22 ± 0.04	83.50	93.00	98.25	99.75
Lower lip (L14)	0.92 ± 0.04	94.25	97.75	98.75	99.75
Subnasale (L15)	1.15 ± 0.05	87.00	90.00	91.50	96.75
Soft tissue pogonion (L16)	1.23 ± 0.06	83.50	90.75	94.50	98.00
Posterior nasal spine (L17)	0.96 ± 0.05	94.00	95.50	96.75	97.75
Anterior nasal spine (L18)	1.49 ± 0.07	77.00	82.50	87.75	93.25
Articulare (L19)	1.43 ± 0.08	76.75	83.75	88.75	94.50
Average	1.20 ± 0.06	84.70	89.38	92.62	96.30

**Table 3:**
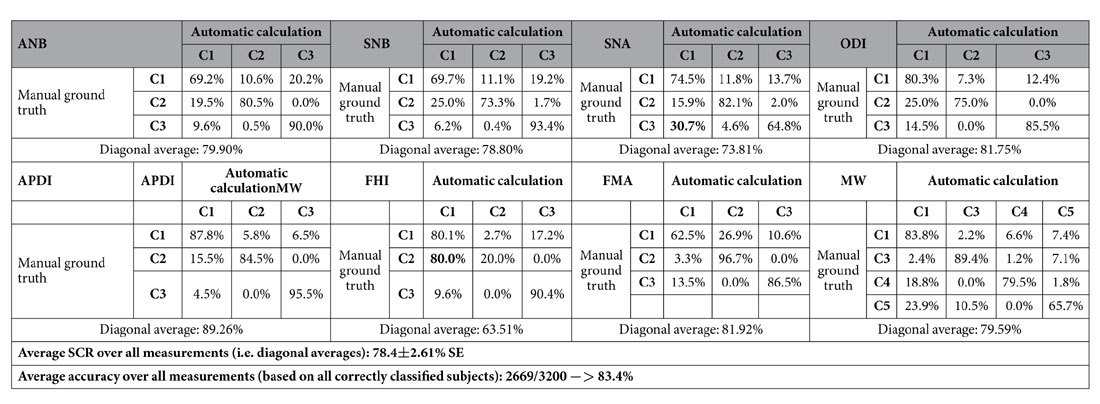
Confusion matrices of the automatic doctor2 (i.e. automatically identified landmark positions by FALA system) vs the doctor2 ground truth (i.e. manually placed landmark positions) classifications for eight clinical measurements to diagnose skeletal malformations in 400 subjects. Diagonals give successful classification rates (SCR).

**Table 4:**
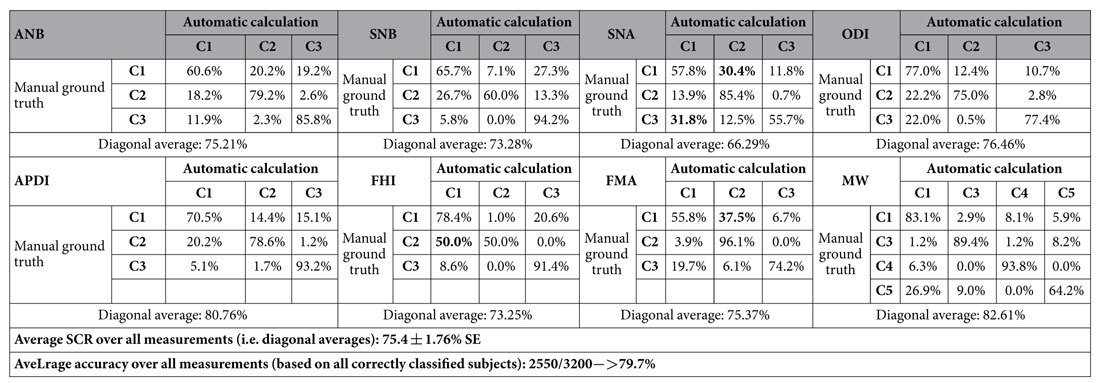
Confusion matrices of the classifications based on the manually placed doctor1 vs the manually placed doctor2 ground truth annotations for eight clinical measurements to diagnose skeletal malformations in 400 subjects.

Diagonals give successful classification rates (SCR).
