# Author Correction: Fully Automatic System for Accurate Localisation and Analysis of Cephalometric Landmarks in Lateral Cephalograms

**DOI:** 10.1038/s41598-021-91681-7

**Published:** 2021-06-02

**Authors:** Claudia Lindner, Ching-Wei Wang, Cheng-Ta Huang, Chung-Hsing Li, Sheng-Wei Chang, Tim F. Cootes

**Affiliations:** 1 Centre for Imaging Sciences, The University of Manchester, Oxford Road, M13 9PT Manchester, United Kingdom; 2 Graduate Institute of Biomedical Engineering, National Taiwan University of Science and Technology, Taipei, Taiwan; 3 NTUST Centre of Computer Vision and Medical Imaging, National Taiwan University of Science and Technology, Taipei, Taiwan; 4 Department of Information Management, Oriental Institute of Technology, Taipei, Taiwan; 5 Orthodontics and Dentofacial Orthopedics Division, Dental Department, Tri-Service General Hospital, Taipei, Taiwan; 6 School of Dentistry & Graduated Institute of Dental Science, National Defense Medical Center, Taipei, Taiwan

Correction to: *Scientific Reports* 10.1038/srep33581, published online 20 September 2016

The Acknowledgements section in this Article is incomplete.

“C. Lindner is funded by the Engineering and Physical Sciences Research Council, UK (EP/M012611/1). C. Wang is supported by the Ministry of Science and Technology, Taiwan (MOST104-2221-E-011-085). C. Huang is funded by the Ministry of Science and Technology, Taiwan (MOST104-2811-E-011-027).”

should read:

“C. Lindner is funded by the Engineering and Physical Sciences Research Council, UK (EP/M012611/1). C. Wang is supported by the Ministry of Science and Technology, Taiwan (MOST104-2221-E-011-085). C. Huang is funded by the Ministry of Science and Technology, Taiwan (MOST104-2811-E-011-027). This work was partially supported by Tri-Service General Hospital-National Taiwan University of Science and Technology (TSGH-NTUST-103-02).”

