# Supplementary material for: Fully Automatic System for Accurate Localisation and Analysis of Cephalometric Landmarks in Lateral Cephalograms
Source: Sci Rep. 2016 Sep 20;6:33581. doi: 10.1038/srep33581 (PMC5028843; doi:10.1038/srep33581)
Supplement: Supplementary Information [file srep33581-s1.pdf]

Fully Automatic System for Accurate Localisation and Analysis of Cephalometric Landmarks in Lateral Cephalograms

Claudia Lindner, Ching-Wei Wang, Cheng-Ta Huang, Chung-Hsing Li, Sheng-Wei Chang, Tim F. Cootes

SUPPLEMENTARY MATERIAL

Table S1

Quantitative comparison of the average point-to-point errors (in pixels, 1 pixel = 0.1mm) for all methods submitted to the ISBI 2014 and ISBI 2015 Grand Challenges on automated cephalometric landmark detection. All methods were tested on the same dataset of 100 cephalograms. Results also include the statistics of a paired sample T-test conducted in SPSS between the best achieving method (7) and each of the other methods demonstrating that (7) is significantly better (p <0.0001). Results reproduced under CC BY 4.0 licence from http://dx.doi.org/10.1016/j.media.2016.02.004.

**Method**	**N**	**Mean**	**Std. deviation**	**Std. error**
Chen (1)	100	28.4722	7.69665	0.76966
Chu (2)	100	26.7896	7.39018	0.73902
Ibragimov (3)	100	19.191	6.18062	0.61806
Mirzaalian (4)	100	23.5309	8.4784	0.84784
Vandaele (5)	100	21.9842	9.11095	0.9111
Ibragimov (6)	100	18.507	6.35577	0.63558
Lindner (7)	100	16.5614	5.39071	0.53907

Paired sample T-test	*Mean*	*Std. deviation*	*t*	*Sig. (2-tailed)*
Chen (1) – Lindner (7)	11.91	5.63	21.138	<0.0001
Chu (2) – Lindner (7)	10.23	5.59	18.313	<0.0001
Ibragimov (3) – Lindner (7)	2.63	4.56	5.769	<0.0001
Mirzaalian (4) – Lindner (7)	6.97	6.96	10.017	<0.0001
Vandaele (5) – Lindner (7)	5.42	7.21	7.519	<0.0001
Ibragimov (6) – Lindner (7)	1.95	4.66	4.173	<0.0001

References:

1. Chen, C., Wang, C.W., Huang, C.T., Li, C.H., and Zheng, G. *Fully-Automatic Landmark detection in Skull X-Ray images*. Automatic Cephalometric X-Ray Landmark Detection Challenge (ISBI 2014). http://www-o.ntust.edu.tw/~cweiwang/celph/paper/chen.pdf
2. Chu, C., Chen, C., Wang, C.W., Huang, C.T., Li, C.H., Nolte, L.P, and Zheng, G. *Fully automatic cephalometric x-ray landmark detection using random forest regression and sparse shape composition*. Automatic Cephalometric X-Ray Landmark Detection Challenge (ISBI 2014). http://www-o.ntust.edu.tw/~cweiwang/celph/paper/chu.pdf
3. Ibragimov, B., Likar, B., Pernus, F., and Vrtovec, T. *Automatic cephalometric X-ray landmark detection by applying game theory and random forests*. Automatic Cephalometric X-Ray Landmark Detection Challenge (ISBI 2014). http://www-o.ntust.edu.tw/~cweiwang/celph/paper/bulat.pdf
4. Mirzaalian, H. and Hamarneh, G. *Automatic globally-optimal pictorial structures with random decision forest based likelihoods for cephalometric x-ray landmark detection*. Automatic Cephalometric X-Ray Landmark Detection Challenge (ISBI 2014). http://www-o.ntust.edu.tw/~cweiwang/celph/paper/mirzaalian.pdf
5. Vandaele, R., Maree, R., Jodogne, S., and Geurts, P. *Automatic cephalometric x-ray landmark detection challenge 2014: A tree-based approach*. Automatic Cephalometric X-Ray Landmark Detection Challenge (ISBI 2014). http://www-o.ntust.edu.tw/~cweiwang/celph/paper/vandaele.pdf
6. Ibragimov, B., Likar, B., Pernus, F. and Vrtovec, T. *Computerized Cephalometry by Game Theory with Shape- and Appearance-Based Landmark Refinement*. Grand Challenges in Dental X-ray Image Analysis – Automated Detection and Analysis for Diagnosis in Cephalometric X-ray Image (ISBI 2015). http://www-o.ntust.edu.tw/~cweiwang/ISBI2015/challenge1/isbi2015_Ibragimov.pdf
7. Lindner, C. and Cootes, T. *Fully Automatic Cephalometric Evaluation using Random Forest Regression-Voting.* Grand Challenges in Dental X-ray Image Analysis – Automated Detection and Analysis for Diagnosis in Cephalometric X-ray Image (ISBI 2015). http://www-o.ntust.edu.tw/~cweiwang/ISBI2015/challenge1/isbi2015_Lindner-Cootes.pdf

Table S2

Cephalometric landmarks and their description.

ID	Landmark name	Landmark description
L1	Sella	The geometric centre of the pituitary fossa (sella turcica), determined by inspection of a constructed point in the midsagittal plane.
L2	Nasion	The intersection of the internasal and frontonasal sutures, in the midsagittal plane.
L3	Orbitale	A point midway between the lowest points on the inferior margin of the two orbits (eye sockets).
L4	Porion	The central point on the upper margin of the external auditory meatus.
L5	Subspinale (A-point)	The deepest (most posterior) midline point on the curvature between the ANS and prosthion.
L6	Supramentale (B-point)	The deepest (most posterior) midline point on the bony curvature of the anterior mandible, between infradentale and pogonion.
L7	Pogonion	The most anterior point on the contour of the bony chin.
L8	Menton	The most inferior point of the mandibular symphysis.
L9	Gnathion	The most anterior inferior point on the bony chin.
L10	Gonion	The most posterior inferior point on the outline of the angle of the mandible.
L11	Incision inferius	The incisal tip of the most labially placed mandibular incisor.
L12	Incision superius	The incisal tip of the most labially placed maxillary central incisor.
L13	Upper lip	Labrale superior (Ls) The point denoting the vermilion border of the upper lip, in the midsagittal plane.
L14	Lower lip	Labrale inferior (Li) The point denoting the vermilion border of the lower lip, in the midsagittal plane.
L15	Subnasale	The point where the base of the columella of the nose meets the upper lip.
L16	Soft tissue pogonion	The most prominent point on the soft tissue contour of the chin.
L17	Posterior nasal spine	The most posterior point on the bony hard palate (nasal floor).
L18	Anterior nasal spine (ANS)	The tip of the bony anterior nasal spine at the inferior margin of the piriform aperture.
L19	Articulare	A constructed point representing the intersection of three radiographic images: the inferior surface of the cranial base and the posterior outlines of the ascending rami or mandibular condyles.

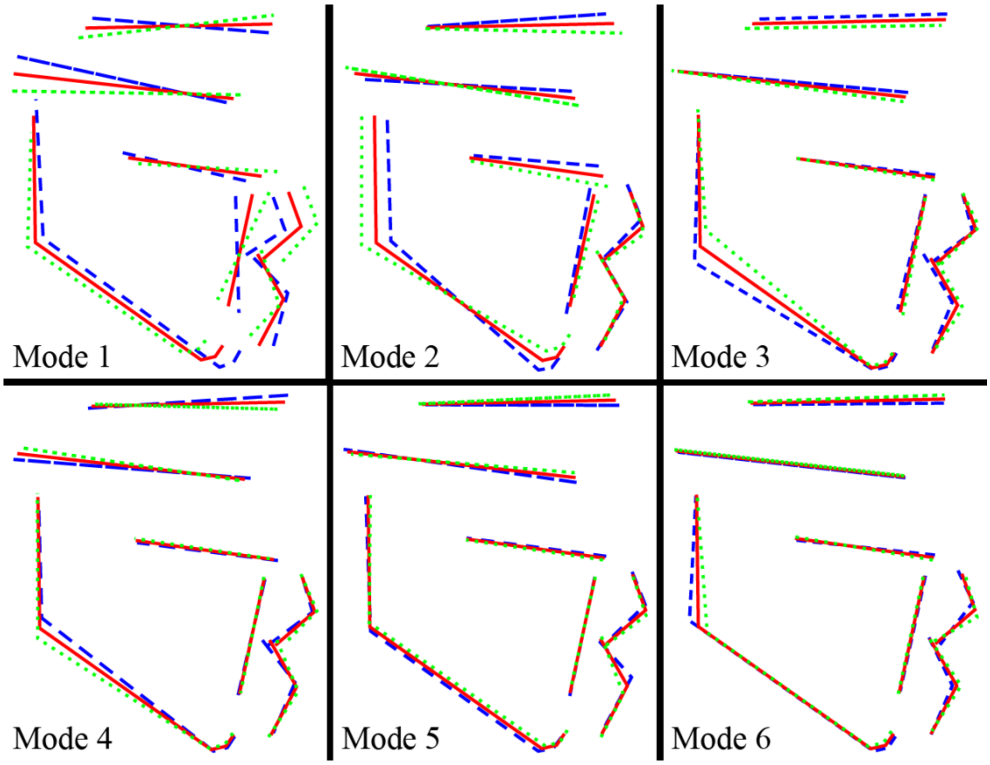

Figure S3: Shape variation exhibited by the first six modes of a statistical shape model (Ref. 26, DOI: http://dx.doi.org/10.1006/cviu.1995.1004) built from the doctor2 ground truth annotations of all 400 images, explaining 75% of the overall shape variation. Each figure shows the average (–) and ±2.0 standard deviations. For easier visualisation of differences, the landmarks were connected as follows: [1-2], [3-4], [5-6], [7-9-8-10-19], [11-14-16], [12-13-15] and [17-18].

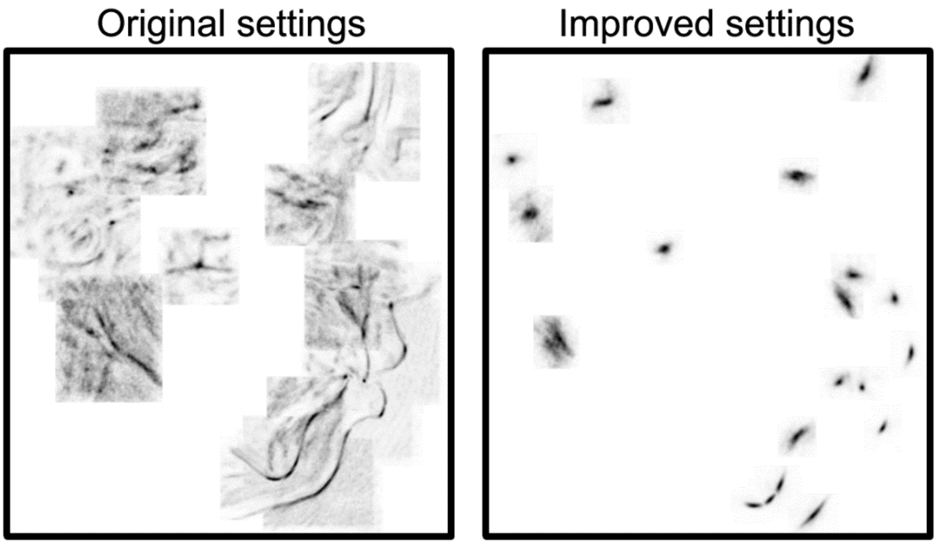

Figure S4: The output of the 19 individual RF landmark predictors on an example test image represented as superposed voting response patches: for (left) the original parameter settings (Ref. 22, DOI: http://dx.doi.org/10.1109/TMI.2013.2258030); and (right) the proposed improved parameter settings. The darker a location is the more likely is the landmark position.

Table S5

Confusion matrices of the automatic doctor1 (i.e. automatically identified landmark positions by FALA system) vs the doctor1 ground truth (i.e. manually placed landmark positions) classifications for eight clinical measurements to diagnose skeletal malformations in 400 subjects. Diagonals give successful classification rates (SCR).

ANB		Automatic calculation		
		C1	C2	C3
Manual ground truth	C1	59.2%	12.6%	28.2%
	C2	37.9%	57.5%	4.6%
	C3	8.1%	1.0%	91.0%
**Diagonal average:**		**69.21%**		

SNB		Automatic calculation		
		C1	C2	C3
Manual ground truth	C1	75.8%	8.4%	15.8%
	C2	20.9%	79.1%	0.0%
	C3	8.0%	1.2%	90.8%
**Diagonal average:**		**81.90%**		

SNA		Automatic calculation		
		C1	C2	C3
Manual ground truth	C1	70.4%	15.5%	14.1%
	C2	25.4%	72.0%	2.7%
	C3	31.9%	0.0%	68.1%
**Diagonal average:**		**70.17%**		

ODI		Automatic calculation		
		C1	C2	C3
Manual ground truth	C1	75.8%	8.6%	15.6%
	C2	36.0%	62.0%	2.0%
	C3	16.5%	0.0%	83.5%
**Diagonal average:**		**73.78%**		

APDI		Automatic calculation		
		C1	C2	C3
Manual ground truth	C1	75.8%	12.1%	12.1%
	C2	18.0%	78.7%	3.4%
	C3	11.2%	0.0%	88.8%
**Diagonal average:**		**81.08%**		

FHI		Automatic calculation		
		C1	C2	C3
Manual ground truth	C1	81.6%	2.0%	16.4%
	C2	60.0%	40.0%	0.0%
	C3	12.7%	0.0%	87.3%
**Diagonal average:**		**69.63%**		

FMA		Automatic calculation		
		C1	C2	C3
Manual ground truth	C1	66.4%	23.1%	10.6%
	C2	11.4%	86.9%	1.7%
	C3	13.6%	0.0%	86.4%
**Diagonal average:**		**79.90%**		

MW		Automatic calculation			
		C1	C3	C4	C5
Manual ground truth	C1	82.7%	0.7%	8.6%	7.9%
	C3	4.7%	89.5%	0.0%	5.8%
	C4	18.8%	0.9%	78.6%	1.7%
	C5	26.9%	9.0%	0.0%	64.2%
**Diagonal average:**		**80.40%**			

Average SCR over all measurements (i.e. diagonal averages): 75.8±1.98% SE

Average accuracy over all measurements (based on all correctly classified subjects): 2552/3200 -> 79.8%
